# Nasal decolonization of *Staphylococcus aureus* and the risk of surgical site infection after surgery: a meta-analysis

**DOI:** 10.1186/s12941-020-00376-w

**Published:** 2020-07-30

**Authors:** Jia Tang, Jiangjin Hui, Jing Ma, Chen Mingquan

**Affiliations:** 1 Department of Infectious Diseases and Hepatology, Huashan Hospital, Fudan University, Shanghai, 200040 China; 2 Department of Infectious Diseases, The First Affiliated Hospital of Zhejiang University, Hangzhou, 310003 China; 3 Department of Endocrinology and Metabolism, Gansu Provincial Hospital, Lanzhou, 730000 China; 4 Department of Emergency, Huashan Hospital, Fudan University, Shanghai, 200040 China

**Keywords:** Nasal decolonization, *Staphylococcus aureus*, Surgical site infections, Meta-analysis

## Abstract

**Aim:**

To assess the effects of nasal decontamination on preventing surgical site infections (SSIs) in people who are *Staphylococcus aureus* carriers undergoing different types of surgeries and diverse measures of decolonization.

**Methods:**

Relevant randomized controlled trials (RCTs) were identified through systematic searches of the PubMed, Embase, Web of science, and the Cochrane Library databases. The risk ratios (RRs) and 95% confidence intervals (CIs) were calculated and the effects model was chosen according to the heterogeneity. Subgroup analyses were performed according to different types of surgeries and measures of decolonization that *Staphylococcus aureus* carriers were applied.

**Results:**

Twenty RCTs published between 1996 and 2019 involving 10,526 patients were included. Pooled results showed that the overall SSIs and pulmonary surgery SSIs presented with a statistical difference in measures of nasal decontamination (RR = 0.59 and 0.47, respectively, both p < 0.01). However, the associations between nasal decolonization and increased risks of SSIs in orthopedics surgery or cardiovascular surgery remained insignificant in studies. As for the diverse measures of nasal decontamination, 50% used mupirocin, 15% used chlorhexidine, 30% used different types of antimicrobial drugs, and 5% use others. The SSIs rate were decreased after chlorhexidine (RR = 0.474, 95% CI 0.259–0.864), while no significant difference was shown in the use of mupirocin (RR = 0.871, 95% CI 0.544–1.394).

**Conclusion:**

It seems that nasal decolonization of *Staphylococcus aureus* may be associated with a reduction of SSIs in these patients, especially in patients who have been administered by pulmonary surgeries or treated with chlorhexidine.

## Introduction

*Staphylococcus aureus* (*S. aureus*), which is normally presented in the microbiota of the human skin and is generally asymptomatic. It remains one of the most common drug-resistant pathogens that causes infection in hospitalized patients [1, 2]. Investments in infection reduction have been posed in intensive care units, which has been defined as an “epicenter” of nosocomial infections, by measurements of skin decolonization involving daily chlorhexidine bathing [3]. The practice was adopted because of evidence that universal decolonization reduces device-associated bacteremia, all-cause bacteremia, and multidrug-resistant organisms [3, 4]. However, the nasal carriage is also unavoidable for endogenous infections and for transmission to other individuals, as the colonization of extra nasal sites often originates from the nasal reservoir [5]. *S. aureus* nasal carriage has been extensively studied by numerous studies, as it was the most common pathogen associated with a postoperative surgical site infections (SSIs), what remains unclear is the exact source of the pathogen [6].

It has been shown that being a nasal carrier of *S. aureus* is a significant risk factor for developing a SSI [7]. In this regard, it seems that the number of SSIs acquired in hospitals may be reduced by decolonization of nasal *S. aureus* carriage on admission [8]. Special attention was paid to nasal decontamination for prevention of SSIs in *S. aureus* carriers. The results of several RCTs in different hospitals and institutions are still mixed and inconclusive, limited by the population and surgery form [6, 9]. The aim of the study is to evaluate the use of nasal decontamination in different types of surgery and provide some evidence that makes efforts to measure of infection control and prevention.

## Methods

This study was performed in accordance with the guideline of Preferred Reporting Items for Systematic Reviews and Meta Analyses [10] and Cochrane’s Handbook [11] guidelines. A prospective protocol was registered in advance and uploaded to the PROSPERO online platform. The registration number is CRD42020170139.

### Literature search

We searched English literature in PubMed, Embase, Web of science, and the Cochrane Library using combinations of the following terms: (1)Nasal or Nose; (2) *Staphylococcus aureus*; (3) Mupirocin or Chlorhexidine or Decontamination. We limited the search to human studies published in randomized trials. All databases were searched from the date of inception up to 20 December 2019. The search strategy used in PubMed is shown in Additional file 1: Appendix S1.

### Eligibility criteria

Studies were eligible for this review if they met the following criteria: (1) a randomized controlled trial of human; (2) had to describe the standard microbial isolation and identification, like *S. aureus*, MRSA or MSSA; (3) included patients scheduled for surgery without infectious diseases. Reviews, case reports, conference abstracts, animal experiments, letters, editorials and studies without randomization were excluded.

### Data extraction and quality evaluation

Data from appropriate studies were pulled out independently by authors and potted into a spreadsheet. Inconsistencies were resolved by unanimity. The data extracted included (1) first author, location, and study design characteristics; (2) procedure characteristics and number, mean age, and gender of patients; (3) methods for screening of nasal *S. aureus* colonization, and strategies for decolonization for nasal MRSA carriers; (4) types of surgery and SSIs; (5) total numbers of *S. aureus* patients and non-colonized patients according to the results of nasal swab examination and the number of patients with SSIs in each group after surgeries. Study quality evaluation was performed with the Cochrane risk of bias tool [11] which includes allocation concealment, blinding, outcome assessment, loss to follow-up (attrition), and the extent of imbalance of the study arms at the beginning of the trial.

### Statistical analyses

We performed a meta-analysis to estimate pooled relative risks (RRs) and 95% confidence intervals (CIs) in STATA version 13.0 (StataCorp, College Station, TX, USA). Heterogeneity was assessed by the I^2^ statistic, for which an I^2^ > 50% suggested substantial heterogeneity and vice versa. In this, I^2^ values of < 25%, 25–50%, 50–75%, and > 75%, were suggestive of low, moderate, substantial, and considerable heterogeneity, respectively. The effects model was chosen according to the heterogeneity. If the I^2^ was ≤ 50%, fixed effect model should be applied while I^2^ > 50% random effect model should be applied. Furthermore, visual assessment of publication bias was shown using the funnel plot. In our study, p < 0.05 was considered a significant difference.

## Results

### Study selection

**Fig. 1:**
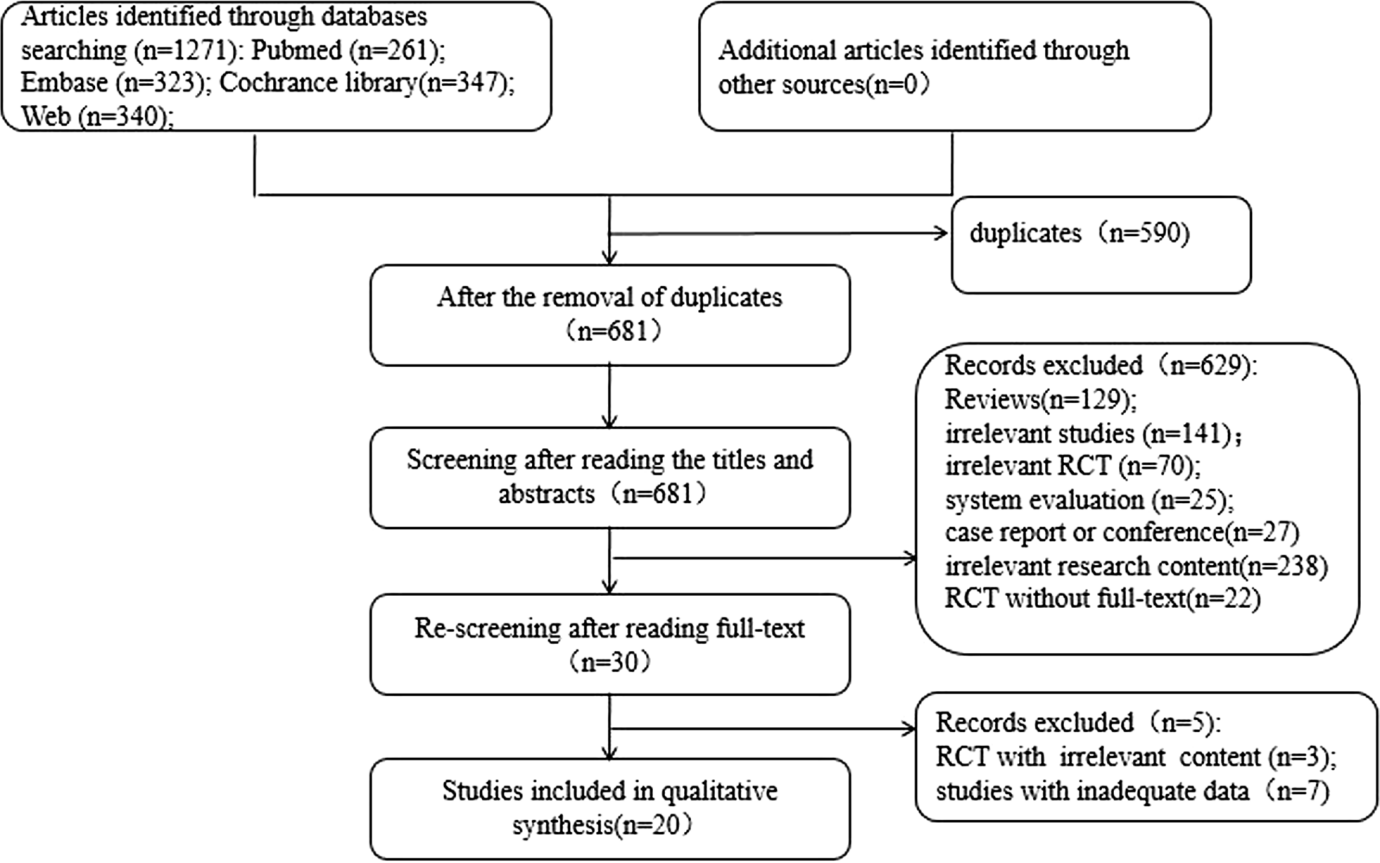
Flow chart

The study selection process is presented in Fig. 1. A total of 1271 relavent studies were searched. 681 studies were subjected to abstract review, excluding many reviews, letters, conference abstracts, editorials and laboratory studies. The remaining 30 studies were subjected to full-text review to exclude those with irrelevant subjects or those that did not fully meet the inclusion criteria. Ultimately, 20 studies were included in the meta-analyses [12–31].

### Study characteristics and quality assessment

**Fig. 2:**
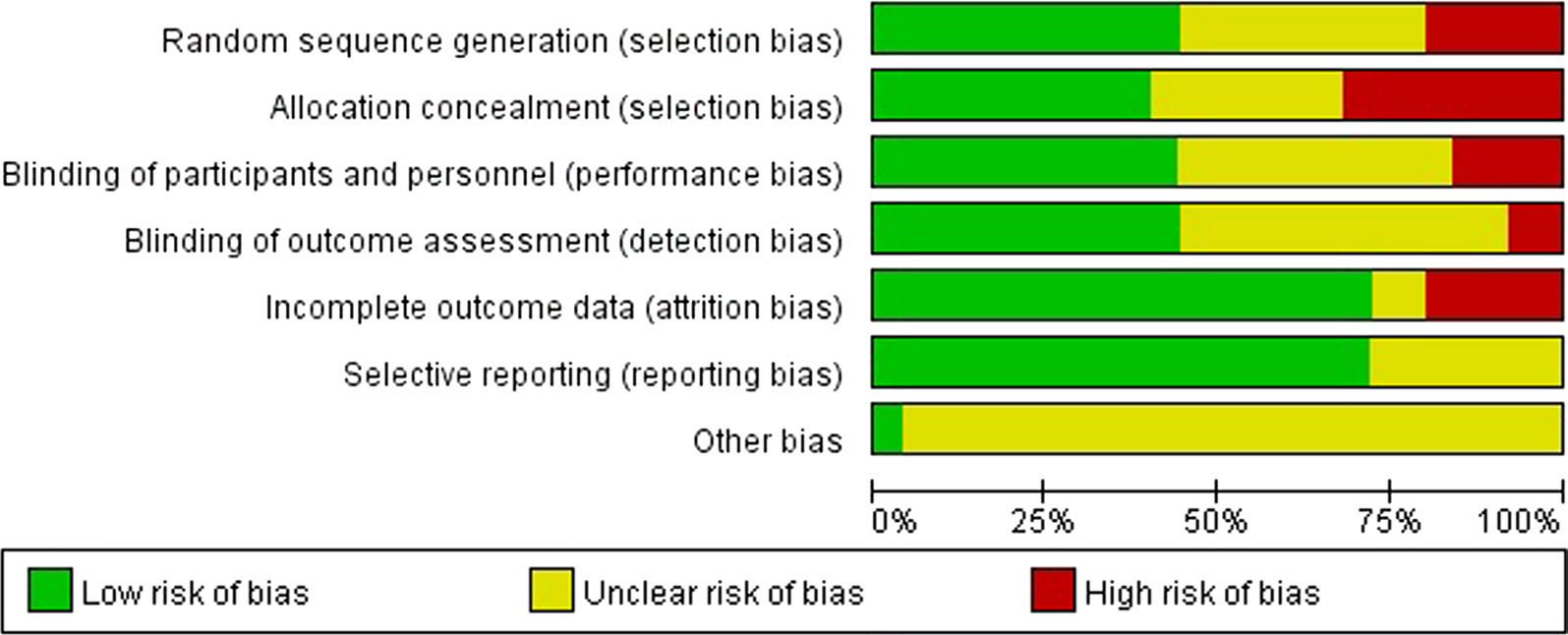
The quality assessment of included articles

The characteristics of the included studies are presented in Table 1. The studies were published between 1996 and 2019 and performed in the United States [14, 17, 18, 21, 24, 26], Netherlands [16, 28–30], and Australia [19, 23, 31]. Besides, the quality of included articles according to Cochrane’s Book was shown in Fig. 2.

**Table 1:** Characteristics of the included studies

	First author	Year	Country	Design	Types of surgery	Number	Age	Intervention
1	Akira	2006	Japan	RCT	Endoscopic gastrostomy	48	73–74	Mupirocin, arbekacin, and sulfamethoxazole/trimethoprim vs untreated
2	Xavier	2018	Germany	RCT	Lung cancer surgery	450	49–9	Chlorhexidine gluconate (CHG) vs placebo
3	Laura	2014	American	RCT	Lung cancer surgery	365	55.5–77.9	Chlorhexidine vs untreated
4	Guy	2016	Israel	RCT	Cesarean section	568	26.8–37.4	Mupirocin vs control
5	Albertine	1998	Netherlands	RCT	Orthopedics	100	18 vs 10	Mupirocin vs control
6	Michael	2014	American	RCT	Arthroplasty or spine fusion	1697	19.1–93.2	Mupirocin vs iodine
7	Nalini	2008	American	RCT	Joint arthroplasty	1377	NA	Mupirocin vs TJA
8	Helena	2018	Australia	RCT	Dermatological closures	142	55.2–77.4	Cephalexin vs placebo
9	Saleh	2016	Sweden	RCT	Dermatological closures	40	45-92	PHMB-based solution vs sterile water
10	Shuman	2012	American	RCT	Head and neck surgery	84	57.5–58.14	Topical antimicrobial decolonization vs standard prophylaxis alone
11	Talesh	2017	Iran	RCT	Head and neck surgery	44	19.7–45.3	Mupirocin vs untreated
12	Yee	2013	Australia.	RCT	Mohs micrographic surgery	738	64–67	Mupirocin vs untreated
13	Berg	2004	American	RCT	Cardiac surgery	296	54.4–72.2	Clarithromycin vs placebo
14	Konvalinka	2006	Canada	RCT	Cardiac surgery	257	51.7–73.3	Mupirocin vs placebo
15	Zibari	1997	American	RCT	Thrombectomized grafts surgery	408	17–81	Vancomycin vs not vancomycin
16	Andenaes	1996	Norway	RCT	Orthopedics	339	24	Azithromycin vs placebo
17	Bode	2016	Netherlands	RCT	Cardiac surgery	793	NA	Mupirocin vs placebo
18	Kalmeijer	2002	Netherlands	RCT	Orthopedics	614	48.1–77.3	Mupirocin vs placebo
19	Kluytmans	1998	Netherlands	RCT	Lung cancer surgery	816	NA	Chlorhexidine vs placebo
20	Smith	2019	Australia	RCT	Mohs micrographic surgery	1350	51–81	Mupirocin vs untreated

### Nasal decolonization and the risk of overall SSIs after surgery

**Fig. 3:**
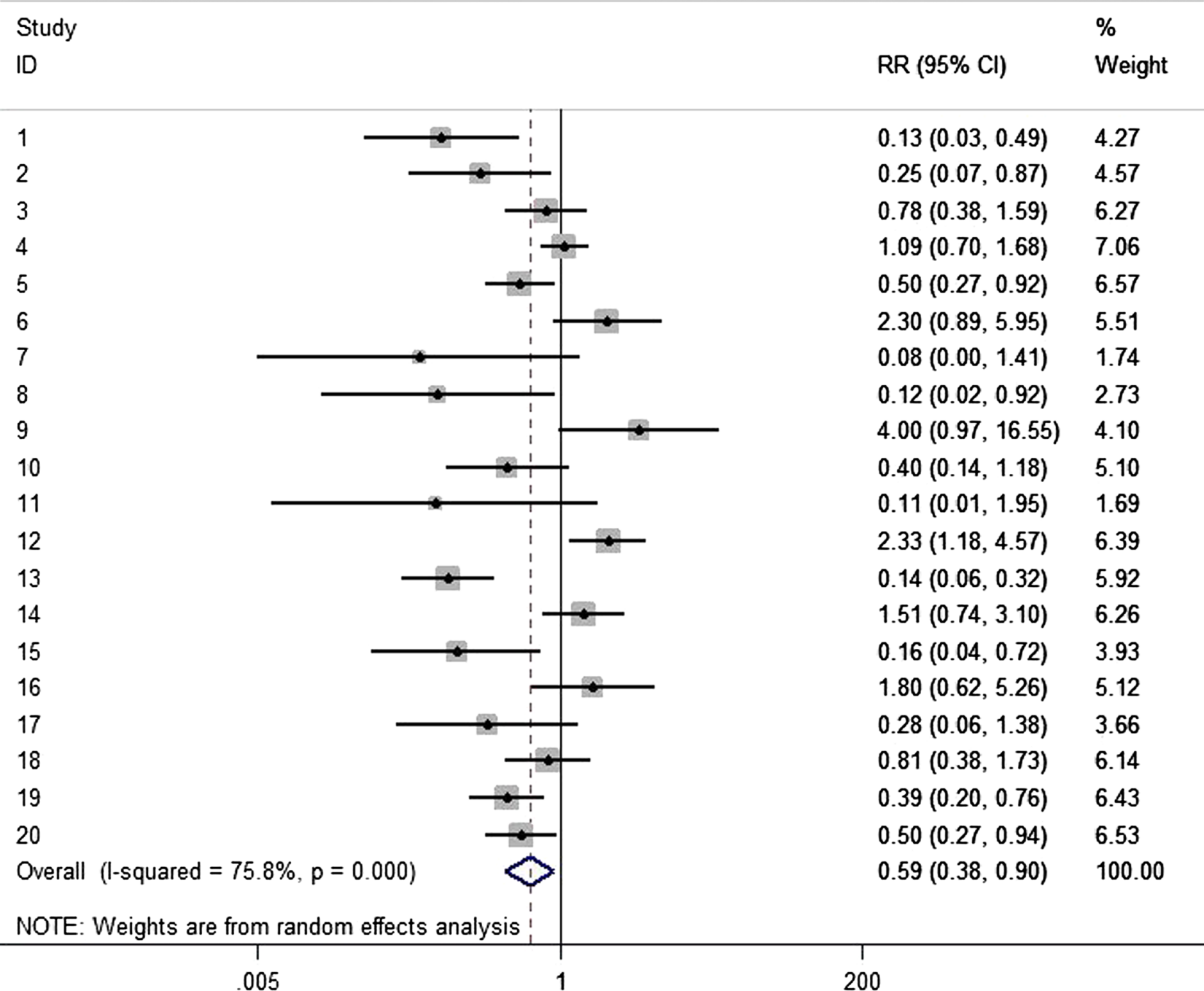
Nasal decolonization and the risk of overall SSI after surgery

The pooled results from 20 studies [12–31] accounting of 10,526 patients showed that nasal decolonization may be associated with a significantly decreased risk of overall SSI in patients after surgery (RR = 0.59, 95% CI 0.38–0.90; Fig. 3). Random effects model was chosen to balance the statistical heterogeneity (p for Cochrane’s Q test = 0.000, I^2^ = 75.8%). Thus, further subgroups analyses were posed to illustrate specific relationships.

### Nasal decolonization and the risk of SSIs after orthopedics surgery

**Fig. 4:**
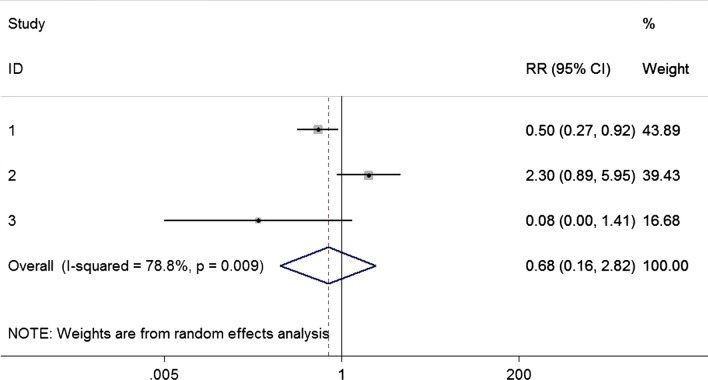
Nasal decolonization and the risk of SSI after orthopedics surgery

Three articles [16–18] with 3174 patients reported the SSIs and orthopedics surgery, showing that there was no statistical difference (RR = 0.68, 95% CI 0.16–2.82; Fig. 4). The substantial heterogeneity (p for Cochrane’s Q test = 0.009, I^2^ = 78.8%) was demonstrated with analysis of random model.

### Nasal decolonization and the risk of SSIs after cardiovascular surgery

**Fig. 5:**
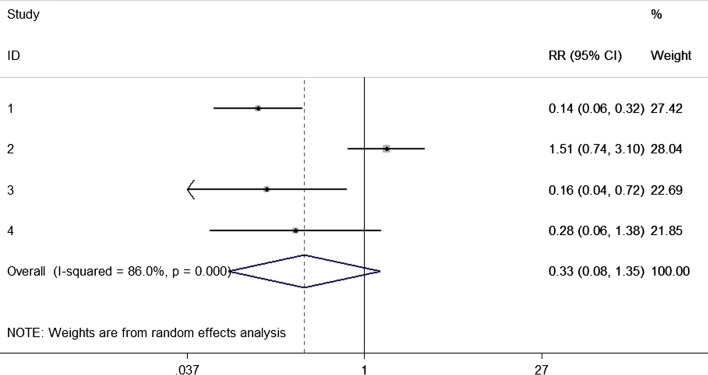
Nasal decolonization and the risk of SSI after cardiovascular surgery

Four studies [24–28] enrolled 1754 patients comparing the decolonization in cardiovascular surgeries. No statistical difference was detected in the meta-analysis (RR = 0.33, 95% CI 0.08–1.35; Fig. 5). Statistical heterogeneity (p for Cochrane’s Q test = 0.000, I^2^ = 86.0%) was handled in random model.

### Nasal decolonization and the risk of SSI after pulmonary surgery

**Fig. 6:**
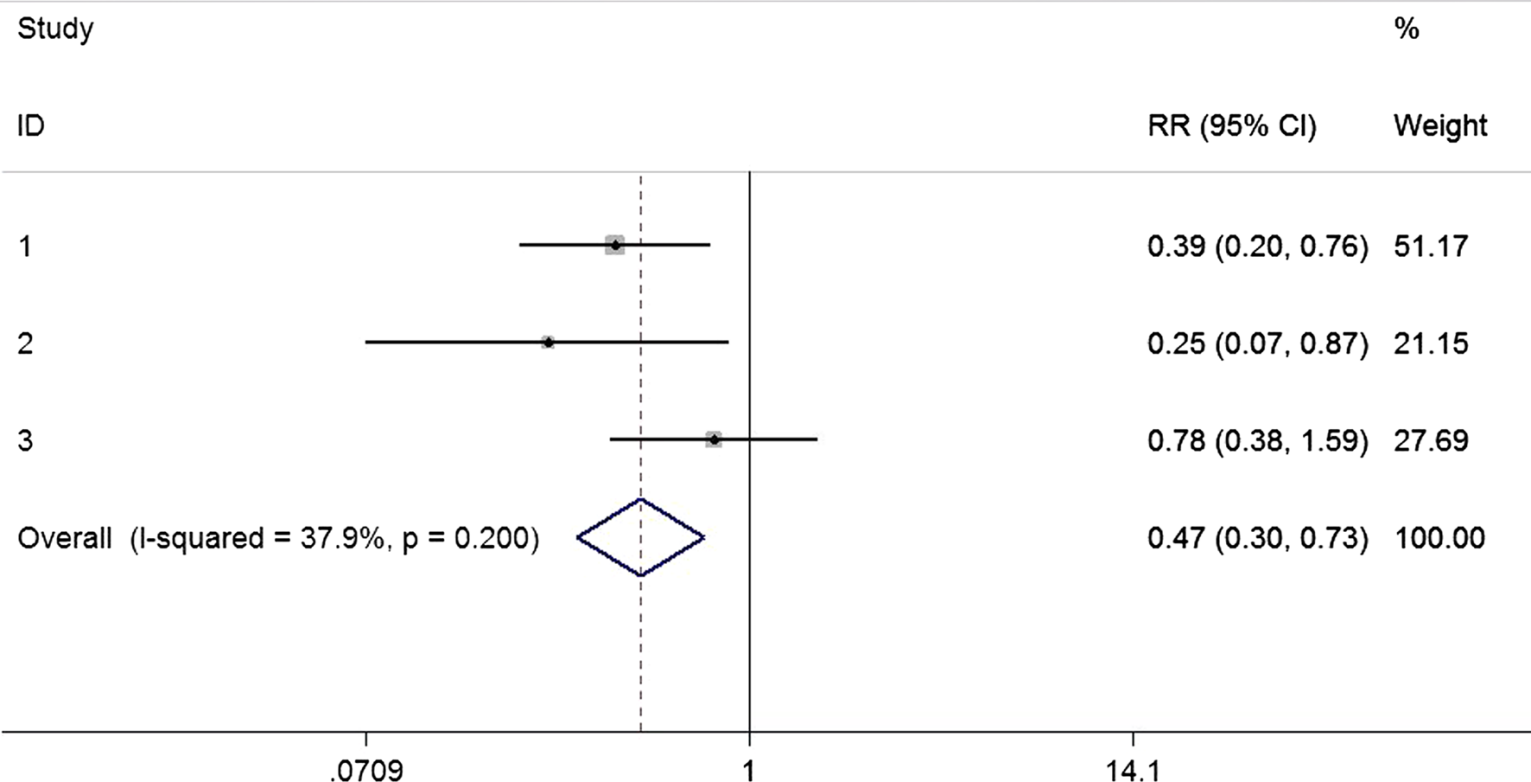
Nasal decolonization and the risk of SSI after pulmonary surgery

Pooled estimates from three studies [13, 14, 30] presented that nasal decolonization related to a significantly decreased risk of SSI in patients after pulmonary surgery (RR = 0.47, 95% CI 0.30–0.73; Fig. 6). Moreover, no significant heterogeneity was detected (p for Cochrane’s Q test = 0.20, I^2^ = 37.9%).

### Nasal decolonization and the risk of SSI with different interventions

**Fig. 7:**
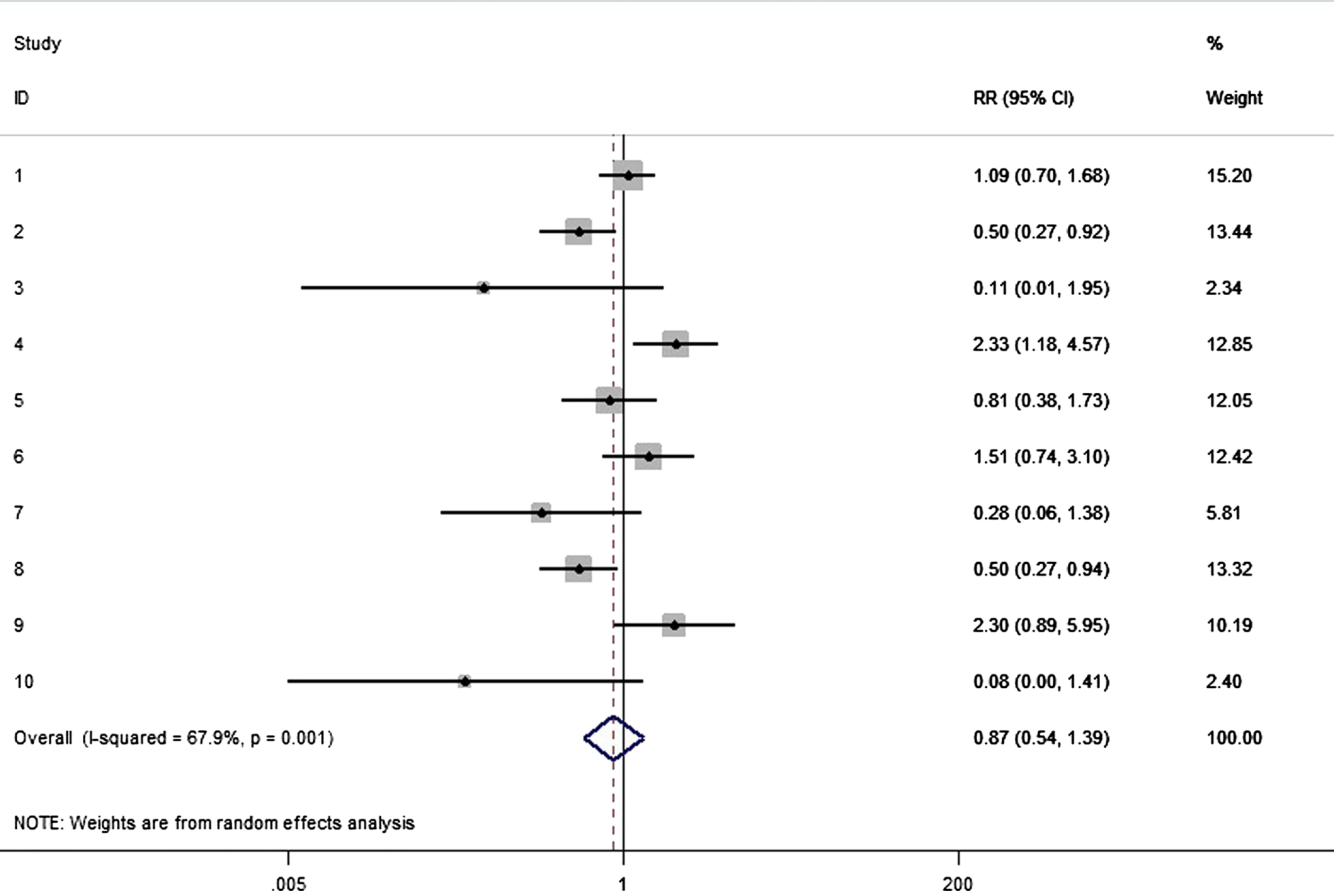
Nasal decolonization and the risk of SSI with mupirocin

**Fig. 8:**
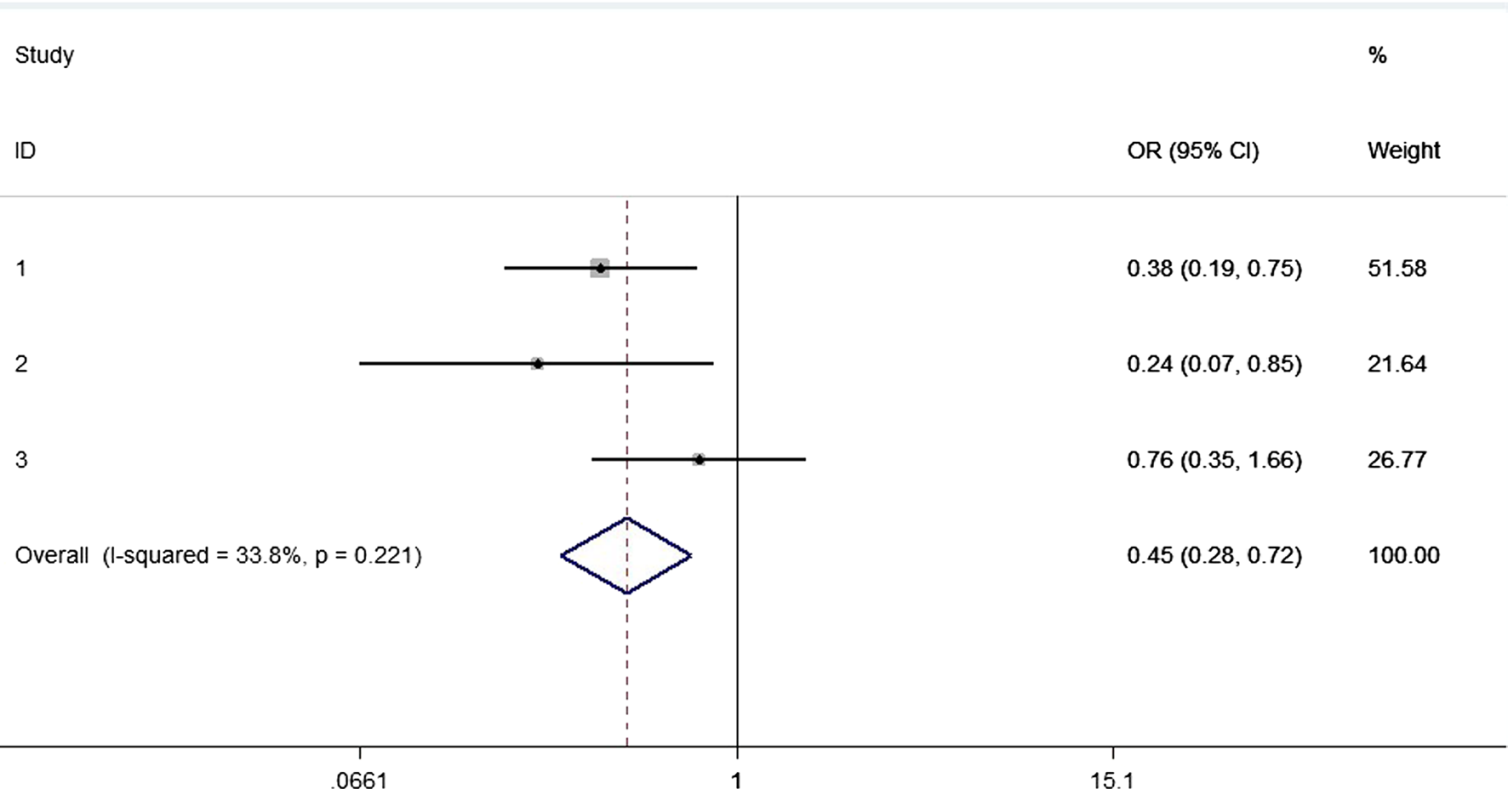
Nasal decolonization and the risk of SSI with chlorhexidine

The interventions of decolonization were diverse, showing that 50% used mupirocin, 15% used chlorhexidine, 30% used different types of antimicrobial drugs, and 5% use others. As for that, ten articles comparing mupirocin with untreated administration, no significant difference was concluded (RR = 0.87, 95% CI 0.54–1.39; Fig. 7), while the heterogeneity was detected and balanced through random model (p for Cochrane’s Q test = 0.33, I^2^ = 67.9%). However, there was a decreased risk of SSI in patients after chlorhexidine, with statistical difference (RR = 0.45, 95% CI 0.28–0.72; Fig. 8), and no significant heterogeneity was detected (p for Cochrane’s Q test = 0.22, I^2^ = 33.8%).

### Publication bias

Funnel plots for the associations between nasal *S. aureus* decolonization and overall SSI risks were shown in Additional file 2: Appendix S2. The funnel plots were symmetric on visual inspection.

## Discussion

Our systematic review has identified important gaps in the literature on targeted decolonization strategies in *Staphylococcus aureus* carriers with different types of surgery. The overall SSIs and pulmonary surgery SSIs presented with a statistical difference in measures of nasal decontamination.

In an early meta-analysis of two randomized trials in cardiac surgery patients, limited by the number of studies, the results showed that no clear difference in SSI risk following the use of mupirocin compared with placebo (RR 1.60, 95% CI 0.79 to 3.25) [9, 25]. Moreover, a recent meta-analysis [32] reported that nasal MRSA colonization may be associated with increased risks of overall SSI and MRSA-SSI after spine surgeries through seven studies (RR = 2.52 and 6.21, respectively, both p < 0.001). Furthermore, a prospective, randomized, single-blinded trial, mentioned about SSIs after elective orthopedic surgery, found that no difference in the risk of SSI between the decolonization and control groups in 1318 patients, both in *S. aureus* carriers and non-carriers [33]. Different results could also be found in another RCT [3], Huang et al. found decolonization with universal chlorhexidine bathing and targeted mupirocin for MRSA carriers did not significantly reduce multidrug-resistant organisms in non-critical-care patients. In light of this, more prospective, randomized-controlled, multi-center studies were needed to articulate the relationships among them.

Trojan Horse [34] claimed a hypothesis trying to explain SSI pathogenesis, showing that pathogens remote from the SSI area—such as within the teeth, noses, or gastrointestinal tract—can be taken up by immune cells (macrophages or neutrophils) and travel to the wound site where they cause wound infections. This mechanism could be verified in a mice model, which can also explain why some infections occur latently following surgery and are due to organisms not found in the wound at the end of the operation [6, 35].

Several limitations derived from this systematic review must be acknowledged. First, the number of included randomized studies was small, which prevented us from evaluating the potential influences of strains of *S. aureus* (e.g. hospital-associated MRSA/MSSA, community-associated MRSA/MSSA) on the association between nasal *S. aureus* colonization and SSIs events. In addition, some studies involved in this study are in high risk and high heterogeneity, which might result in inevitable bias. Last but not least, the adverse effects of decolonization (e.g. increased risk of drug resistance) had been merely mentioned, which is significant for an appliance.

To conclude, the main pillars of from available evidence, it seems that nasal decontamination may be associated with a reduction of overall SSIs in patients with pulmonary surgery or treated with chlorhexidine. Further studies are needed to validate and propose the specific relationships between host and infection.

## Supplementary information

**Additional file 1.** Search strategy.

**Additional file 2.** Funnel plot.

## Data Availability

All data generated or analysed during this study are included in this published article and its additional files.

## Abbreviations

SSIs: Surgical site infections
RCTs: Randomized controlled trials
RRs: Risk ratios
CIs: Confidence intervals
*S. aureus*: *Staphylococcus aureus*
MRSA: Methicillin-resistant *Staphylococcus aureus*
MSSA: Methicillin-sensitive *Staphylococcus aureus*
