# Supplementary material for: Nasal decolonization of Staphylococcus aureus and the risk of surgical site infection after surgery: a meta-analysis
Source: Ann Clin Microbiol Antimicrob. 2020 Jul 30;19:33. doi: 10.1186/s12941-020-00376-w (PMC7392830; doi:10.1186/s12941-020-00376-w)
Supplement: Supplementary file 1 — Additional file 1. Search strategy. [file 12941_2020_376_MOESM1_ESM.doc]

# Appendix1 Search Strategy

**Pubmed**

1 "Nose"[Mesh](89388)

2 nose*[Title/Abstract] OR nas*[Title/Abstract] OR rhin*[Title/Abstract](222709)

3 OR/1‐2 (263236)

4 "Staphylococcal Infections"[Mesh](64329)

5 "Staphylococcus aureus"[Mesh](72484)

6 staphylococ*[Title/Abstract] OR "S aureus"[Title/Abstract] (151246)

7 OR/4-6 (179213)

8 "Anti-Infective Agents"[Mesh](688597)

9 "Antibiotic Prophylaxis"[Mesh](13469)

10 "Mupirocin"[Mesh](1201)

11 "Chlorhexidine"[Mesh](8141)

12 "Povidone-Iodine"[Mesh](2767)

13 "Administration, Intranasal"[Mesh](14263)

14 "Decontamination"[Mesh](4699)

15 antibiotic*[Title/Abstract] OR antimicrobial*[Title/Abstract] OR antibacterial*[Title/Abstract] OR antiseptic*[Title/Abstract])(495123)

16 Mupirocin[Title/Abstract] OR Chlorhexidine[Title/Abstract] OR Povidone-Iodine[Title/Abstract] OR bactroban[Title/Abstract] OR centany[Title/Abstract] OR eismycin[Title/Abstract] OR plasimine[Title/Abstract] OR "pseudomonic acid"[Title/Abstract] OR Naseptin[Title/Abstract] OR CHG[Title/Abstract](14681)

17 intranasal*[Title/Abstract] OR decontamin*[Title/Abstract] OR decoloni*[Title/Abstract](38945)

18 OR/8-17(986952)

19 3 AND 7 AND 18 (3123)

20 19 AND ("randomized controlled trial"[pt] OR "controlled clinical trial"[pt] OR "randomized controlled trials as topic"[mh] OR "clinical trials as topic"[mh] OR "controlled clinical trials as topic"[mh] OR placebos[mh] OR "random allocation"[mh] OR "double-blind method"[mh] OR randomized[tiab] OR placebo[tiab] OR randomization[tiab] OR randomly allocated [tiab] OR ((double[tw] OR treble[tw] or triple[tw]) AND (mask*[tw] OR blind* [tw])))(261)

**Ovid Embase**

1 ‘Nose’/exp(71488)

2 ‘nose*’.ti,ab,kw OR ‘nas*’.ti,ab,kw OR ‘rhin*’.ti,ab,kw (378968)

3 OR/1-2(395367)

4 ‘Staphylococcal Infections’/exp(53200)

5 ‘Staphylococcus aureus’/exp(164414)

6 staphylococ*.ti,ab,kw OR ‘S aureus’.ti,ab,kw(187094)

7 OR/4-6(257100)

8 ‘Anti-Infective Agents’/exp(3548629)

9 ‘Antibiotic Prophylaxis’/exp(30396)

10 ‘Mupirocin’/exp(6817)

11 ‘Chlorhexidine’/exp(16926)

12 ‘Povidone-Iodine’/exp(10054)

13 ‘Administration, Intranasal’/exp(1962)

14 ‘Decontamination’/exp(13659)

15 antibiotic*.ti,ab,kw OR antimicrobial*.ti,ab,kw OR antibacterial*.ti,ab,kw OR antiseptic*.ti,ab,kw (656851)

16 Mupirocin.ti,ab,kw OR Chlorhexidine.ti,ab,kw OR ‘Povidone-Iodine’.ti,ab,kw OR bactroban.ti,ab,kw OR centany.ti,ab,kw OR eismycin.ti,ab,kw OR plasimine.ti,ab,kw OR ‘pseudomonic acid’.ti,ab,kw OR Naseptin.ti,ab,kw (18203)

17 intranasal*.ti,ab,kw OR decontamin*.ti,ab,kw OR decoloni*.ti,ab,kw(50709)

18 OR/8-17(3741665)

19 3 AND 7 AND 18(6213)

20 19 AND (‘controlled clinical trail’/de OR ‘randomized controlled trial’/de OR "randomized controlled trial (topic)’/de) (323)

**Cochrane library**

1 Mesh descrisiptor : [Nose](2632)

2 nose*.ti,ab,kw OR nas*.ti,ab,kw OR rhin*.ti,ab,kw(39860)

3 OR/1‐2 (40151)

4 Mesh descrisiptor : [Staphylococcal Infections](1167)

5 Mesh descrisiptor : [Staphylococcus aureus](781)

6 staphylococ*.ti,ab,kw OR ‘S aureus’.ti,ab,kw (5486)

7 OR/4-6 (5545)

8 3 AND 7 (671)

9 Mesh descrisiptor : [Anti-Infective Agents](28226)

10 Mesh descrisiptor : [Antibiotic Prophylaxis](1240)

11 Mesh descrisiptor : [Mupirocin](2056)

12 Mesh descrisiptor : [Chlorhexidine](588)

13 Mesh descrisiptor : [Povidone-Iodine](2587)

14 Mesh descrisiptor : [Administration, Intranasal](76)

15 Mesh descrisiptor : [Decontamination](205)

16 antibiotic*.ti,ab,kw OR antimicrobial*.ti,ab,kw OR antibacterial*.ti,ab,kw OR antiseptic*.ti,ab,kw(21)

17 Mupirocin.ti,ab,kw OR Chlorhexidine.ti,ab,kw OR Povidone-Iodine.ti,ab,kw OR bactroban.ti,ab,kw OR centany.ti,ab,kw(5569)

18 eismycin.ti,ab,kw OR plasimine.ti,ab,kw OR ‘pseudomonic acid’.ti,ab,kw OR Naseptin.ti,ab,kw OR CHG.ti,ab,kw(339)

19 intranasal*.ti,ab,kw OR decontamin*.ti,ab,kw OR decoloni*.ti,ab,kw(7701)

20 OR/9-19(39779)

21 8 AND 20 (358)

22 21 AND ‘Trials’ (347)

**Web of Science**

#1 主题:[Nose] OR主题:[nose*] OR主题:[nas*] OR主题:[rhin*] (347894)

主题:[] OR

#2 主题:[‘Staphylococcal Infections’] OR 主题:[‘Staphylococcus aureus’] OR 主题:[staphylococ*] OR 主题:[‘S aureus’](164305)

#3 主题:[‘Anti-Infective Agents’] OR 主题:[‘Antibiotic Prophylaxis’] OR 主题:[Mupirocin] OR 主题:[Chlorhexidine] OR 主题:[Povidone-Iodine] OR 主题:[‘Administration, Intranasal’] OR 主题:[Decontamination] (728172)

#4主题:[antibiotic*] OR 主题:[antimicrobial*] OR 主题:[antibacterial*] OR 主题:[antiseptic*] (528721)

#5 主题:[Mupirocin] OR 主题:[Chlorhexidine] OR 主题:[Povidone-Iodine] OR 主题:[bactroban] OR 主题:[centany] OR 主题:[eismycin] OR 主题:[plasimine] OR 主题:[‘pseudomonic acid’] OR 主题:[Naseptin] OR 主题:[CHG](36141)

#6主题:[intranasal*] OR 主题:[decontamin*] OR主题:[decoloni*](53535)

#7 #3 OR #4 OR #5 OR #6(1222689)

#8 #1 AND #2 AND #7(3579)

#9 主题:[randomized controlled trial] OR 主题:[controlled clinical trial] OR 主题:[randomized controlled trials as topic] OR 主题:[controlled clinical trials as topic] OR主题:[clinical trials as topic] OR主题:[placebos] OR主题:[random allocation] OR主题:[double-blind method] OR主题:[randomized] OR主题:[placebo] OR主题:[randomization] OR主题:[randomly allocated](834274)

#10主题:[double] OR主题:[treble] OR主题:[triple] (1352767)

#11主题:[mask*] OR 主题:[blind*] (570291)

#12 #10 AND #11(259972)

#13 #12 OR #9(1015613)

#14 #8 AND #13(340)
