# Supplementary figures and images for: Nasal decolonization of Staphylococcus aureus and the risk of surgical site infection after surgery: a meta-analysis
Source: Ann Clin Microbiol Antimicrob. 2020 Jul 30;19:33. doi: 10.1186/s12941-020-00376-w (PMC7392830; doi:10.1186/s12941-020-00376-w)

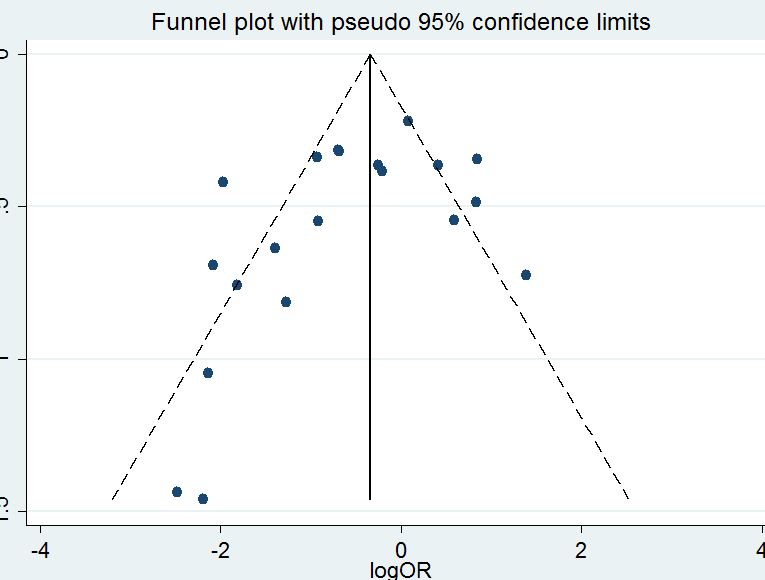

Supplement: Supplementary file 2 — Additional file 2. Funnel plot. [file 12941_2020_376_MOESM2_ESM.jpg]
